# A Unique Branching Pattern of the Brachial Artery: Coexisting Superficial Ulnar Artery and Persistent Median Artery

**DOI:** 10.7759/cureus.29882

**Published:** 2022-10-03

**Authors:** Benjamin J Herstam, Sai Pidatala, Yun Tan, Daniel T Daly

**Affiliations:** 1 Department of Physical Therapy, Doisy College of Health Sciences, Saint Louis University, St. Louis, USA; 2 Department of Surgery, Center for Anatomical Science and Education, Saint Louis University School of Medicine, St. Louis, USA

**Keywords:** brachial artery, embryology, upper limb, anatomical variation, superficial ulnar artery, persistent median artery

## Abstract

The presence of both a superficial ulnar artery (SUA) and persistent median artery (PMA) of antebrachial type is of both clinical and surgical significance. In an 84-year-old female cadaver received through the Gift Body Program at Saint Louis University School of Medicine, the right brachial artery was seen divided into an SUA and radial artery (RA) slightly below the interepicondylar line of the humerus. At the level of the radial neck, the RA sent out the common interosseous artery that then gave off the radial recurrent artery before bifurcating into anterior interosseous artery (AIA) and posterior interosseous artery. The AIA continued to appear to branch into the anterior ulnar recurrent artery and posterior ulnar recurrent artery, as well as a PMA of the antebrachial type. In the hand, the SUA and RA contributed to the complete superficial palmar arch seemingly equally, and the RA was the dominant contribution to the deep palmar arch. Ninety-one other arms were assessed for this variation, and none were observed. Knowledge of an anatomical variation such as this may lead to decreased complications in the planning of surgical bypass grafting.

## Introduction

Typically, the brachial artery (BA) is a continuation of the axillary artery (AA), beginning at the inferior border of teres major and continuing until its bifurcation into the radial artery (RA) and ulnar artery (UA) at the level of the radial neck. The UA typically gives off the common interosseous artery (CIA), which gives rise to the anterior interosseous artery (AIA) and posterior interosseous artery (PIA), as well as the anterior ulnar recurrent artery (AURA) and posterior ulnar recurrent artery (PURA). The UA then continues across the wrist as the main contributor to the superficial palmar arch (SPA) and minor contributor to the deep palmar arch (DPA). The RA typically gives rise to the radial recurrent artery (RRA) and continues across the wrist as the main contribution to the DPA and a minor anastomosis to the SPA. Prior reports outlining variations in the branching of the BA and the antebrachial arterial network show variability; however, the variation reported herein appears to be a novel finding.

Proposed mechanisms of variations in antebrachial and palmar vascular branching stem from fetal development. One such theory outlining arterial development from an axial artery may offer insights into the formation of the persistent median artery (PMA). The axial system develops in the middle of the fourth week of embryonic development and gives rise to the AA, BA, and AIA [1,2]. From the AIA, the median artery develops as a major blood supply to the developing hand [3]. After the eighth week of development when the definitive RA and UA are formed, the median artery commonly undergoes regression and apoptosis [4]. On the contrary, if the median artery does not regress, it can persist into adulthood, representing a supernumerary arterial remnant of the vascular architecture of the axial artery, known as the PMA. The PMA can be of palmar or antebrachial type [4]. The palmar type of PMA crosses the wrist to supply blood to the hand and is larger in caliber than the antebrachial type [5]. The antebrachial type is a delicate remnant of a partial regression of the median artery that terminates proximal to the wrist in the antebrachium while serving as the primary blood supply to the median nerve [5]. Previous reports indicate a PMA prevalence of 4.2%-6.6% with the antebrachial PMA making up nearly 75% of those cases [3-11].

In typical development, the RA and UA are derived from either the AA, BA, or superficial brachial artery. An embryological superficial ulnar artery (SUA) concurrently develops from the BA, then anastomoses with the deeper UA, whereby the superficial artery regresses, resulting in the formation of a definitive UA. However, if the blood flow of the embryologic SUA is greater than that of the embryologic deeper UA, the superficial artery may not undergo apoptosis and regress, which results in the superficial artery persisting as the SUA into adulthood [2]. Thus, the SUA can be defined as a product of axial system development and courses superficially to the antebrachial musculature [12,13]. Previous reports regarding the SUA show a prevalence of 0.17%-2% [13]. Reports of a PMA and an SUA concurrently persisting into adulthood have not been found in the literature. 

This case report presents a novel combination of the presence of both an SUA and a PMA of antebrachial type. The SUA and RA continued to the palm to form a complete type A co-dominant SPA where both the superficial branch of the radial artery (SBRA) and the UA had a matched contribution to the SPA and the RA had a greater contribution to the DPA than did the deep palmar branch of the ulnar artery (DPUA) [14].

## Case presentation

During routine dissection of the right antebrachial region in an 84-year-old female cadaver, a novel variation involving the BA and its branches was found. Upon examination of the left upper extremity of the same female cadaver, a typical branching pattern was noted. Forty-five additional cadavers were assessed for this variation, but none was found, giving this variation a prevalence of 1.09%. All bodies were received through the Saint Louis University Gift of Body Program and the Center for Anatomical Science and Education (CASE) with signed informed consent from the donors. The CASE Gift Body Program abides by all rules set forth by the Uniform Anatomical Gift Act.

**Figure 1:**
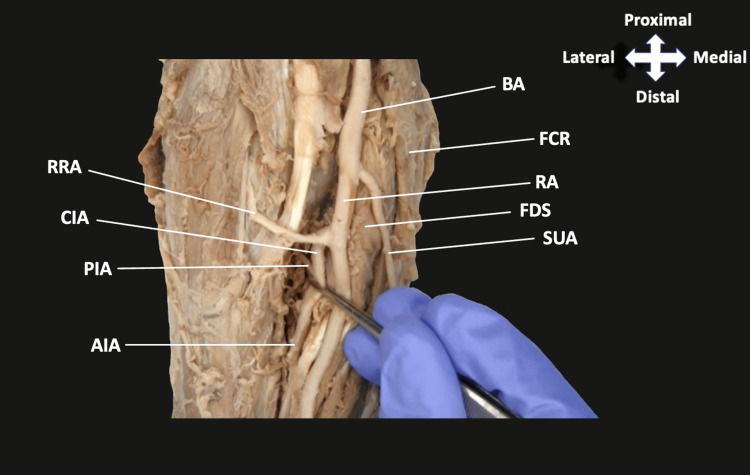
Branching of the BA at the cubital fossa The BA divides into the RA and SUA. The RA then gives off the CIA, while the SUA descends medially. BA, brachial artery; FCR, flexor carpi radialis; RA, radial artery; FDS, flexor digitorum superficialis; SUA, superficial ulnar artery; RRA, radial recurrent artery; CIA, common interosseous artery; PIA, posterior interosseous artery; AIA, anterior interosseous artery

**Figure 2:**
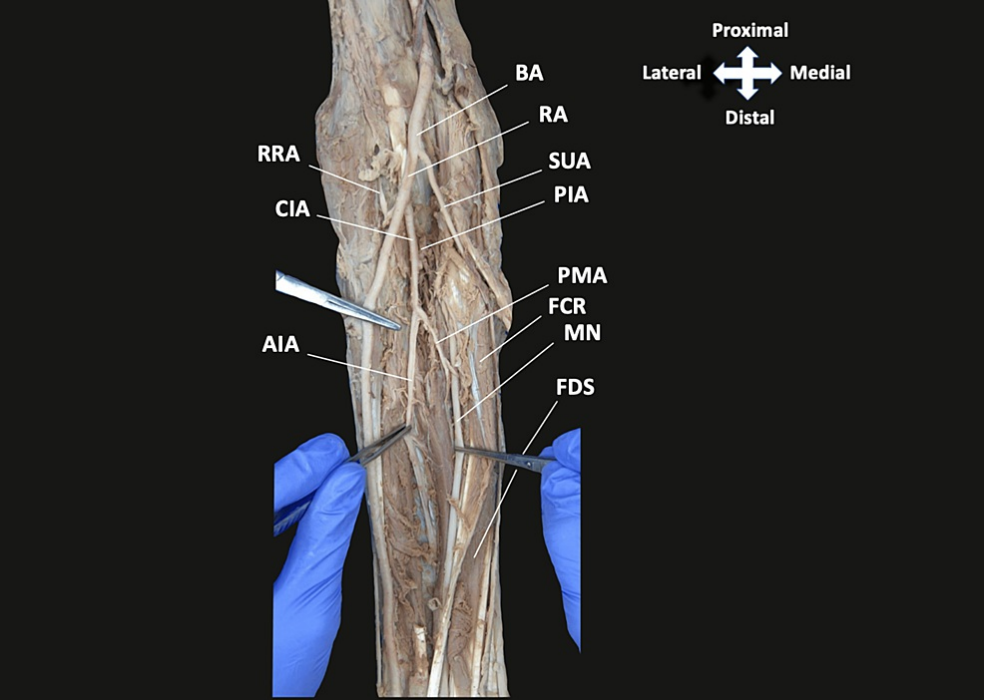
Arterial branching in the antebrachium The CIA descends from the RA giving off the PIA and AIA. The PMA then branches from the AIA and travels alongside the median nerve terminating proximal to the wrist. The SUA continues distally in the medial forearm. BA, brachial artery; RA, radial artery; SUA, superficial ulnar artery; PIA, posterior interosseous artery; PMA, persistent median artery; FCR, flexor carpi radialis; MN, median nerve; FDS, flexor digitorum superficialis; RRA, radial recurrent artery; CIA, common interosseous artery; AIA, anterior interosseous artery

Following the removal of skin and superficial fascia, the BA was noted to branch into a large RA and a small SUA immediately below the interepicondylar line of the humerus (Figures 1, 2). The SUA traveled superficially to the forearm flexor musculature originating from the medial epicondyle and coursed between the flexor digitorum profundus (FDP) and flexor carpi ulnaris distally (Figure 2). The RA gave off the CIA that ran deep to the two heads of the pronator teres muscle (Figure 1). The CIA then branched into the RRA, PIA, and AIA (Figure 1). The AIA then appeared to give off the AURA and PURA, although severed during routine dissection, along with a vessel identified as a PMA of antebrachial type. This PMA traveled with the median nerve and gave a single muscular branch to the flexor digitorum superficialis muscle before continuing distally with the MN (Figure 2).

**Figure 3:**
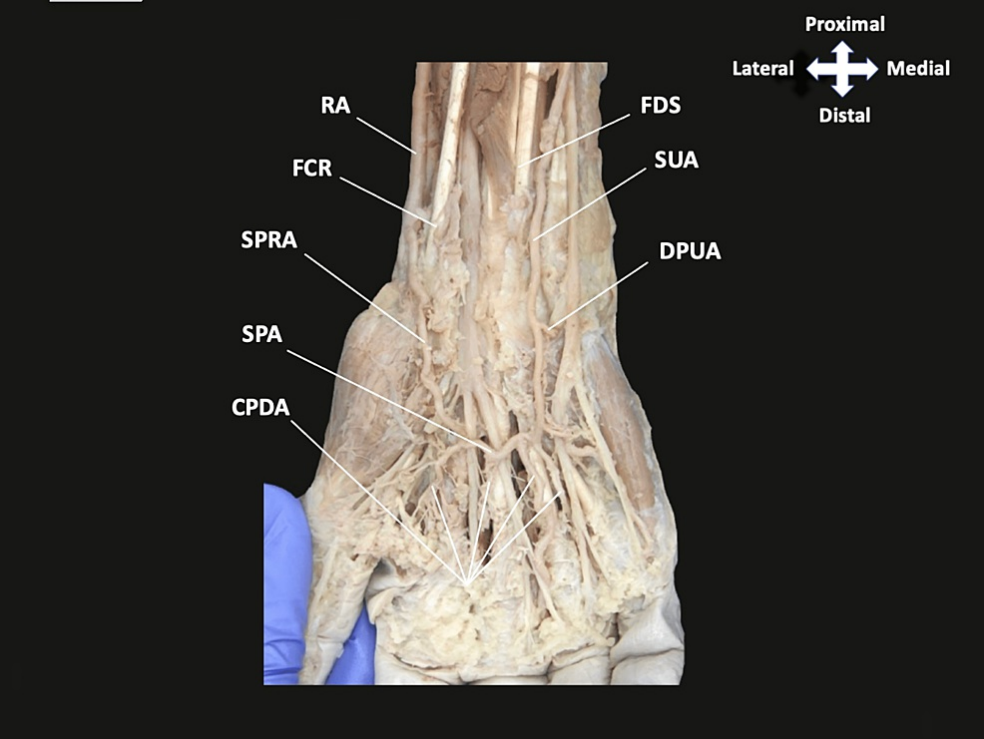
Dissection of the SPA in the hand The RA and SUA descend the antebrachium to contribute to the SPA. As expected, the SUA gave off the common palmar digital arteries. FDS, flexor digitorum superficialis; SUA, superficial ulnar artery; DPUA, deep palmar branch of the ulnar artery; RA, radial artery; FCR, flexor carpi radialis; SPRA, superficial palmar branch of the radial artery; SPA, superficial palmar arch; CPDA, common palmar digital artery

**Figure 4:**
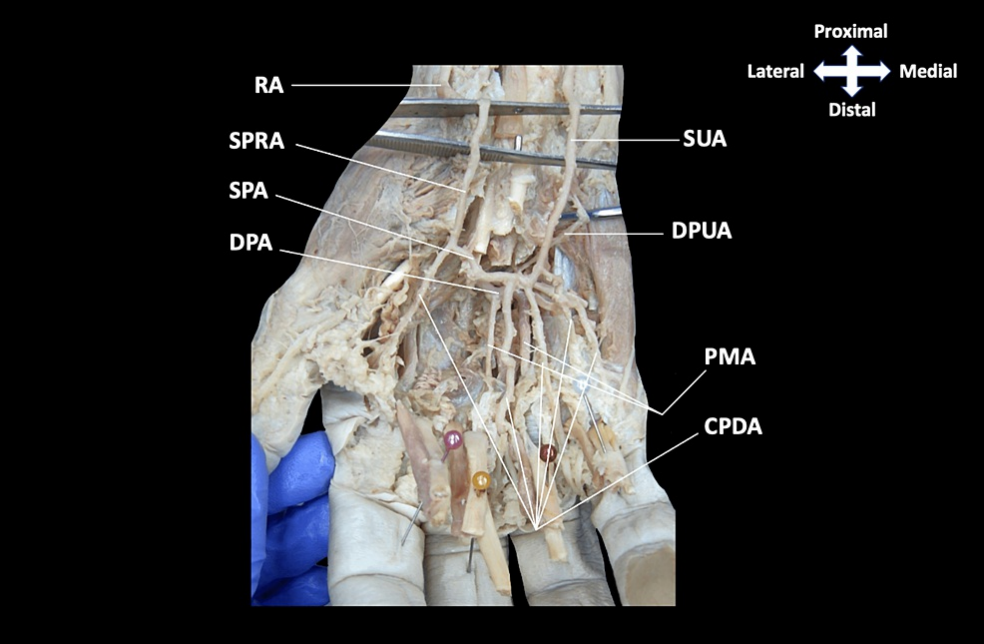
Dissection of superficial and deep arterial arches of the hand Tendons of flexor digitorum profundus, flexor digitorum superficialis, and lumbrical muscles reflected distally, and flexor digiti minimi were removed to visualize the DPUA. The SUA was observed as the minor contribution to the DBA through the DPUA. The DPA gave off the palmar metacarpal arteries as expected, and can be seen more distally located than typically reported, appearing on level with the SPA. SUA, superficial ulnar artery; DPUA, deep palmar branch of the ulnar artery; PMA, palmar metacarpal artery; CPDA, common palmar digital artery; RA, radial artery; SPRA, superficial palmar branch of the radial artery; SPA, superficial palmar arch; DPA, deep palmar arch

As dissection continued distally into the palm, a complete SPA was formed, essentially equally, by an anastomosis between the SUA and the superficial palmar branch of the RA (Figure 3). Typical branching of the common and proper palmar digital arteries was observed from the SPA (Figures 3, 4). Reflection of the tendons of flexor digitorum superficialis, flexor digitorum profundus, and lumbrical muscles was performed to reveal a DPA that was formed by an anastomosis between the RA and DPUA, with the RA as the apparent dominant contribution. As expected, the DPA provided the palmar metacarpal arteries that contribute to the circulation of the digits (Figure 4).

## Discussion

The novel combination of the presence of an SUA along with an antebrachial PMA in this upper limb adds a new clinically significant vascular variation to the body of literature. The SUA, which passes superficially to the flexor muscles originating from the medial epicondyle, is highly susceptible to the inadvertent intra-arterial injection. With a prevalence of up to 2%, the evaluation for an SUA may be beneficial to healthcare providers practicing injection or phlebotomy in the antebrachial region [13]. Additionally, the RA is the second most used artery in coronary artery bypass grafting [15]. With an RA caliber as large as that observed in this case report, ligation and harvesting of the radial artery could remove the major blood supply of the hand. If the RA were to be ligated at the level of the cubital fossa, in a case such as that presented here, all arterial branches to the antebrachium would, resultantly, lose their blood supply. Thus, a case can be made for the SUA to be considered in harvesting for bypass grafting in patients demonstrating arterial branching patterns like the one presented in this case study.

The PMA is commonly concurrent with compressive neuropathies of the upper limb. Because of the path of the PMA as it courses alongside the median nerve, it can compress the nearby neural structures such as the anterior interosseous nerve resulting in anterior interosseous nerve syndrome, where the flexor pollicis longus, lateral two tendons of the FDP, and pronator quadratus undergo atrophy and paralysis [16]. Additionally, the palmar type PMA can play an integral role in the development of acute carpal tunnel syndrome. The palmar PMA can undergo thrombosis, as it passes through the carpal tunnel, thereby compressing the closely related median nerve [17].

The PMA is explained as the persistence of the fetal median artery [4]. However, there is a dearth of notable hypotheses outlining why a median artery may persist beyond its typical point of regression, which is thought of to be week 8 of fetal development [4]. Given the outlined development of the SUA, a similar theory for the persistence of a median artery may be reasonable [2]. In the case presented, where both the SUA and PMA are present, the persistence of a median artery may be a compensatory mechanism for the development of an SUA. This is because the SUA is smaller in caliber than the UA, resulting in a decreased blood flow to the hand [18]. Typically, the RA and UA represent the dominant blood supply to the hand, so the need for the median artery may be negated once they fully develop, and the median artery undergoes regression. However, due to the smaller caliber of the SUA, the median artery may have been a more dominant blood supply to the hand, resulting in it not undergoing regression and apoptosis, and a persistence into adulthood.

## Conclusions

This novel case of the presence of both an SUA and a PMA of antebrachial type is of both clinical and surgical significance. Although the variation described may not be the direct cause of any pathological findings, it is a combination of variations that may be of interest to clinicians. Knowledge of an anatomical variation like this may lead to decreased complications in the planning of surgical bypass grafting. In addition, the PMA component of this set of variations may be important when assessing cases of compressive neuropathies. This case report presents a novel addition to the literature describing the arterial network of the brachial artery and its variant branches.
